# Photon-counting computed tomography in esophageal cancer: correlation of iodine concentration with histopathology and treatment response to neoadjuvant radiochemotherapy

**DOI:** 10.1007/s00330-025-11683-1

**Published:** 2025-05-23

**Authors:** Nina Pauline Haag, Pascal Bodin, Jan Robert Kröger, Julius Henning Niehoff, Saher Saeed, Berthold Gerdes, Raihanatou Ina Danebrock, Ulrich Klaus Fetzner, Jan Borggrefe, Andreas Wienke, Alexey Surov

**Affiliations:** 1 Department of Radiology, Neuroradiology and Nuclear Medicine, Johannes Wesling University Hospital, Ruhr University Bochum, Minden, Germany; 2 Siemens Healthineers USA, Princeton, NJ USA; 3 Department of General-, Visceral-, Thoracic-, Pediatric- and Endocrine Surgery, Johannes Wesling University Hospital, Ruhr University Bochum, Minden, Germany; 4 Department of Pathology, Johannes Wesling University Hospital, Ruhr University Bochum, Minden, Germany; 5 Department of Medical Epidemiology, Biometry, and Informatics, University Hospital Halle, Martin Luther University Halle-Wittenberg, Halle, Germany

**Keywords:** Multidetector computed tomography, Esophagus, Esophageal neoplasms, Technology, radiologic, Neoadjuvant therapy

## Abstract

**Objectives:**

Evaluating esophageal cancer (EC) response to neoadjuvant radiochemotherapy (NARC) has been challenging, but photon-counting CT (PCCT) provides multiparametric data, including iodine concentration (IC), which can be utilized for evaluation. This study explored the relationship between IC and histopathological features of EC, assessing its role in predicting NARC responses.

**Materials and methods:**

Of 105 patients with EC, 85 (67 men; mean age 66.0 ± 11.0 years) met the inclusion criteria and underwent PCCT scans during the portal venous phase. Normalized iodine concentration (NIC) was calculated, and tumor characteristics, including stage, grade, and lymphovascular invasion, were analyzed. Statistical analyses included Mann–Whitney U tests, sensitivity, specificity, and area under the curve (AUC) calculations. Interobserver reliability of NIC measurements was assessed.

**Results:**

Interobserver reliability for NIC was excellent (ICC = 0.99 for all tumors, *p* < 0.01). In adenocarcinoma, NIC was lower in good therapy responders (Becker 1a/1b: 0.40 ± 0.13) than poor responders (Becker 2/3: 0.51 ± 0.12, *p* = 0.01). An NIC cutoff ≤ 0.41 predicted good regression (OR = 4.77, *p* = 0.03; AUC = 0.704, sensitivity = 72.2%, specificity 64.7%). Poor response prediction showed moderate accuracy (AUC = 0.662).

**Conclusion:**

NIC values show excellent interobserver agreement and can predict treatment response to NARC in EC, particularly for adenocarcinomas, where lower NIC values are linked to better outcomes. While NIC provides good predictive value, further studies with larger sample sizes are needed to confirm these findings and explore additional factors influencing outcomes.

**Key Points:**

***Question*** *Does photon-counting CT-derived iodine concentration correlate with key histopathological features in esophageal cancer and predict response to neoadjuvant radiochemotherapy?*

***Findings*** *Photon-counting CT–derived normalized iodine concentration in esophageal adenocarcinoma moderately predicted treatment response, despite no correlation with tumor grade or Ki-67*.

***Clinical relevance*** *Normalized iodine concentration values from photon-counting CT, obtained during routine staging exams, offer an objective method for predicting treatment response in esophageal adenocarcinoma, enabling more precise therapy planning and personalized patient management*.

**Graphical Abstract:**

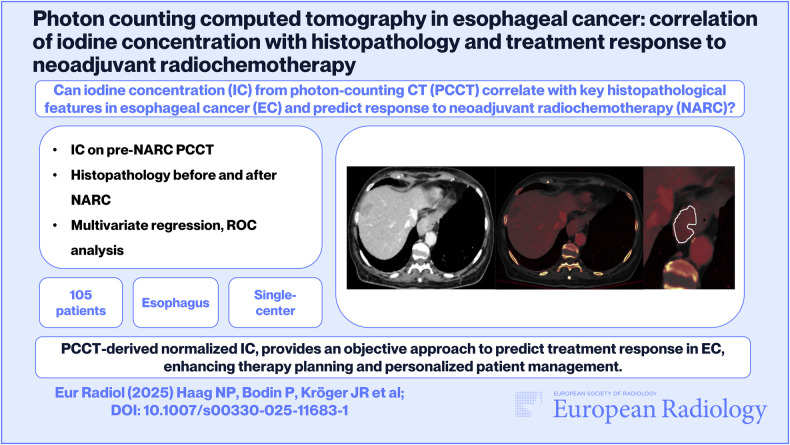

## Introduction

Esophageal cancer (EC) is one of the most common and aggressive malignancies worldwide, with a high mortality rate [1]. Traditionally, diagnostic and staging methods for EC have relied on endoscopic techniques and conventional imaging modalities, such as CT and positron emission tomography [2].

Predicting treatment response is particularly critical in EC, as neoadjuvant radiochemotherapy (NARC) has become the standard therapeutic approach for resectable, locally advanced esophageal adenocarcinoma, while concurrent chemoradiotherapy followed by surgery is recommended for esophageal squamous cell carcinoma (SCC) [2].

Photon-counting computed tomography (PCCT) introduces new possibilities for improving the accuracy of tumor characterization and staging, offering advantages such as reduced electronic noise, improved spatial resolution, and enhanced material decomposition [3, 4]. PCCT’s ability to quantify iodine concentration (IC) within tissues, measured in mg/cm³, presents new opportunities for tumor characterization, to reflect tumor vascularity and treatment response [5–7]. IC has demonstrated utility in differentiating benign from malignant lesions in various organs, such as the parotid gland, and predict treatment response to NARC in rectal cancer [8, 9].

The purpose of the present study is to investigate the association between IC obtained via PCCT and key histopathological features in EC. Furthermore, this study aimed to evaluate the role of IC in predicting tumor response to NARC.

## Materials and methods

### Study design

This retrospective study was approved by the institutional review board (Ethics Committee of the Faculty of Medicine, Ruhr-University Bochum, approval code 2021–827). 105 consecutive Patients diagnosed with locally advanced EC who underwent PCCT as an initial staging scan and a follow-up scan after NARC at our institution between January 1, 2022, and June 30, 2024, were included. The inclusion criteria were histologically confirmed primary esophageal adenocarcinoma or squamous cell carcinoma, and availability of histopathological data, and the execution of a pretreatment PCCT scan of the thorax and abdomen in the portal venous phase. Exclusion criteria included histology types other than adenocarcinoma or squamous cell carcinoma, significant imaging artifacts that compromised interpretation, exams without contrast enhancement, very small tumors (not visible on the CT scan, smaller than 5 mm, with tumor stage T1NxMx, and histological carcinoma in situ), or missing data. Clinical data, including tumor stage (TNM classification), nodal involvement, distant metastasis, and tumor grade were retrieved from the patients’ medical records.

### Photon-counting computed tomography

All patients underwent thorax and abdomen PCCT imaging using a dual-source photon-counting CT scanner (NAEOTOM Alpha, Siemens Healthineers) with software version syngo.CT VA40A and VA50A. Imaging was performed in the portal venous phase after intravenous administration of a weight-adjusted contrast agent (1 mL/kg bodyweight, ACCUPAQUE 300, GE Healthcare) The contrast agent was injected into a peripheral vein at a flow rate of 2.5 mL/s, followed by a saline flush of 40 mL NaCl (0.9%) at 2.5 mL/s. The portal venous phase scan was initiated 60 s after the threshold of 100 HU was detected in the descending aorta. PCCT acquisition parameters were as follows: tube voltage of 120 kVp, image quality level of 170, total collimation of 144 × 0.4 mm, pitch factor of 0.8, and gantry rotation time of 0.5 s.

Each scan included a monoenergetic and a spectral data set reconstructed with specific convolution kernels: Br36 for the monoenergetic data and Qr36 for the spectral data, both utilizing Quantum Iterative Reconstruction at level 4 (Q4). images were generated primarily in the axial plane, with a slice thickness of 1 mm and a 0.4 mm increment, on a 512 × 512 matrix. Iodine maps were generated in a post processing software (syngo.via, VB10, Dual Energy workflow, Siemens Healthineers).

### Iodine concentration analysis

Imaging analysis was performed independently by two radiologists (P.B. with 1 year of experience, and J.H.N., a board-certified radiologist, with 5 years of experience in colorectal imaging). Both radiologists were blinded to the histopathological findings.

The image slice with the largest extension of the tumor was selected, and a polygonal region of interest (ROI) was manually drawn on iodine maps around the margin of the lesion, avoiding partial volume effects. For all tumors, mean IC values were recorded.

Additionally, a circular ROI was placed within the abdominal aorta recoding the mean IC value per case. Normalized iodine concentration (NIC) was calculated as the ratio of the tumor IC to the IC of the aorta to account for physiological variability in iodine accumulation. All image analyses were performed using the manufacturer’s specific software (syngo.via, VB10, Dual Energy workflow, Siemens Healthineers).

### Histopathological analysis

Histopathological features, including tumor cell type, tumor grade, lymphovascular invasion, and venous involvement were assessed on hematoxylin-eosin-stained slides. Additionally, the proliferation index was estimated on Ki-67 antigen-stained specimens by using MIB-1 monoclonal antibody (DakoCytomation Glostrup) as a ratio: number of stained nuclei/(number of all nuclei × 100%). Tumor regression after NARC was graded using the Becker classification, with grades ranging from 1a (no residual tumor) to 3 (poor response) [10]. Histopathological evaluations were performed by a board-certified pathologist with over 20 years of clinical experience.

### Statistical analysis

Statistical analysis was conducted using SPSS (IBM SPSS Statistics for Windows, version 28.0). Continuous variables were expressed as mean values, medians, and standard deviations, while categorical variables were presented as frequencies and percentages. The level of statistical significance was set at *p* < 0.05.

Interobserver reliability was assessed using the intraclass correlation coefficient (ICC) to measure agreement between two independent readers in measuring NIC values. To compare NIC values across different tumor characteristics, such as tumor grade, nodal involvement, and metastatic status, Mann–Whitney U tests were applied. Spearman’s correlation was used to analyze the relationship between NIC values and tumor markers, including Ki-67 and cell count. Correlation coefficients and *p*-values were calculated separately for adenocarcinoma and squamous cell carcinoma. Receiver operating characteristic (ROC) curve analysis was used to evaluate NIC’s predictive performance for tumor response to NARC. Sensitivity, specificity, positive predictive value (PPV), negative predictive value (NPV), and overall accuracy were calculated, and the area under the curve (AUC) was determined. Logistic regression was performed to assess NIC as a potential predictor of treatment response. A multivariate regression analysis was conducted, adjusting for tumor stage, nodal stage, and tumor grade to account for potential confounders. The optimal NIC cutoff for predicting good response was determined based on the maximum Youden index, and odds ratios (OR) with 95% confidence intervals (CI) were calculated.

## Results

### Patient and tumor characteristics

**Fig. 1:**
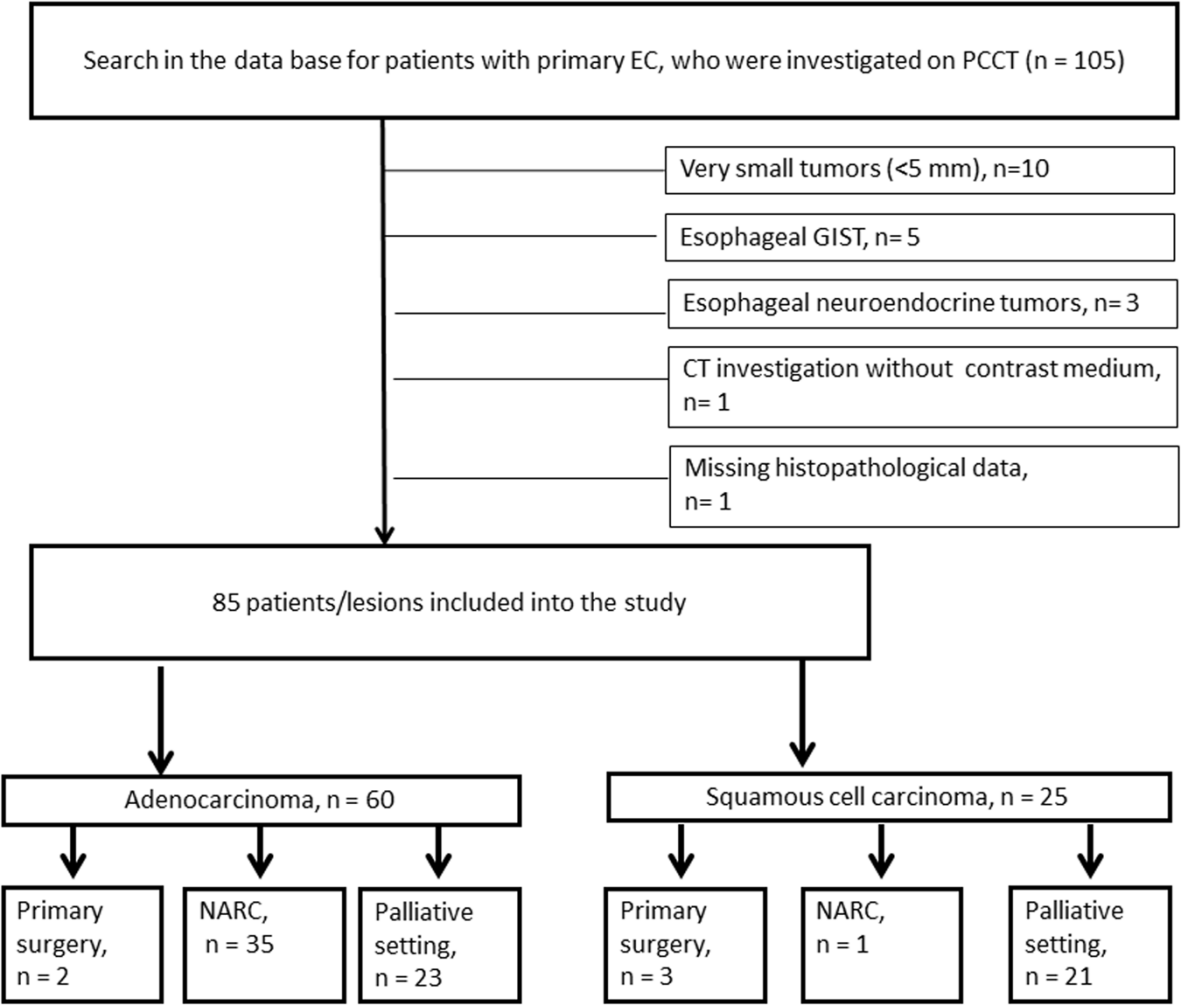
Participant’s flowchart. EC, esophageal cancer; PCCT, photon-counting computed tomography; GIST, gastro-intestinal-stromal-tumor

A total of 105 patients were initially identified, of which 85 met the inclusion criteria. The mean age was 66.0 ± 11.0 years, and the study sample consisted of 67 men and 18 women. Find more detail about the inclusion and exclusion of patients in Fig. 1. Out of all tumors, 58% were adenocarcinomas, among which 18% were classified as T1-T2 stage and 82% as T3-T4 stage, whereas 61% were graded as G3. Nodal involvement (N+) was present in 80%, and distant metastasis (M+) were identified in 25% of all cases.

In the adenocarcinoma subgroup, 35 patients underwent NARC. Additionally, 23 cases presented with advanced, metastatic tumors treated in a palliative setting. Primary surgery was performed in two cases. Treatment responses following NARC in adenocarcinoma were distributed as follows: Becker grade 1a (*n* = 9, 26%), grade 1b (*n* = 9, 26%), grade 2 (*n* = 7, 20%), and grade 3 (*n* = 10, 28%).

For SCC, 21 patients (84%) presented with advanced tumor stages and distant metastases. Primary surgery was performed in three cases, while NARC was administered in only one case. As a result, no analysis of associations between treatment response and NIC could be conducted for this subgroup.

More detail is presented in Table 1.

**Table 1:** Participant characteristics

Characteristic	Value
Male, *n* (%)	67 (79%)
Female, *n* (%)	18 (21%)
Age, years	66.0 ± 11.0
Adenocarcinoma	60
T1-2, *n* (%)	11 (18%)
T3-4, *n* (%)	49 (82%)
N0, *n* (%)	12 (20%)
N+, *n* (%)	48 (80%)
M0, *n* (%)	45 (75%)
M+, *n* (%)	15 (25%)
Grade 1, *n* (%)	1 (2%)
Grade 2, *n* (%)	20 (33%)
Grade 3, *n* (%)	37 (62%)
Grade 4, *n* (%)	1 (2%)
Squamous cell cancer	25
T1-2, *n* (%)	1 (4%)
T3-4, *n* (%)	24 (96%)
N0, *n* (%)	4 (16%)
N+, *n* (%)	21 (85%)
M0, *n* (%)	18 (72%)
M+, *n* (%)	7 (28%)
Grade 1, *n* (%)	0 (0%)
Grade 2, *n* (%)	10 (40%)
Grade 3, *n* (%)	15 (60%)

Values are presented as mean and standard deviation or *n*

*T* tumor stage, *N* nodal involvement, *M* distant metastasis

### Interobserver reliability

The measurements of NIC across different tumor types demonstrated a high degree of reliability between observers. For all tumors combined, the ICC was 0.99 (95% CI = 0.98; 0.99, *p* < 0.01), indicating near-perfect agreement. When analyzed by tumor subtype, NIC measurements showed an ICC of 0.95 (95% CI = 0.90; 0.98, *p* < 0.01) for adenocarcinomas and 0.99 (95% CI = 0.98; 1.00, *p* < 0.01) for squamous cell carcinomas, reflecting consistent reliability across both histological types.

**Fig. 2:**
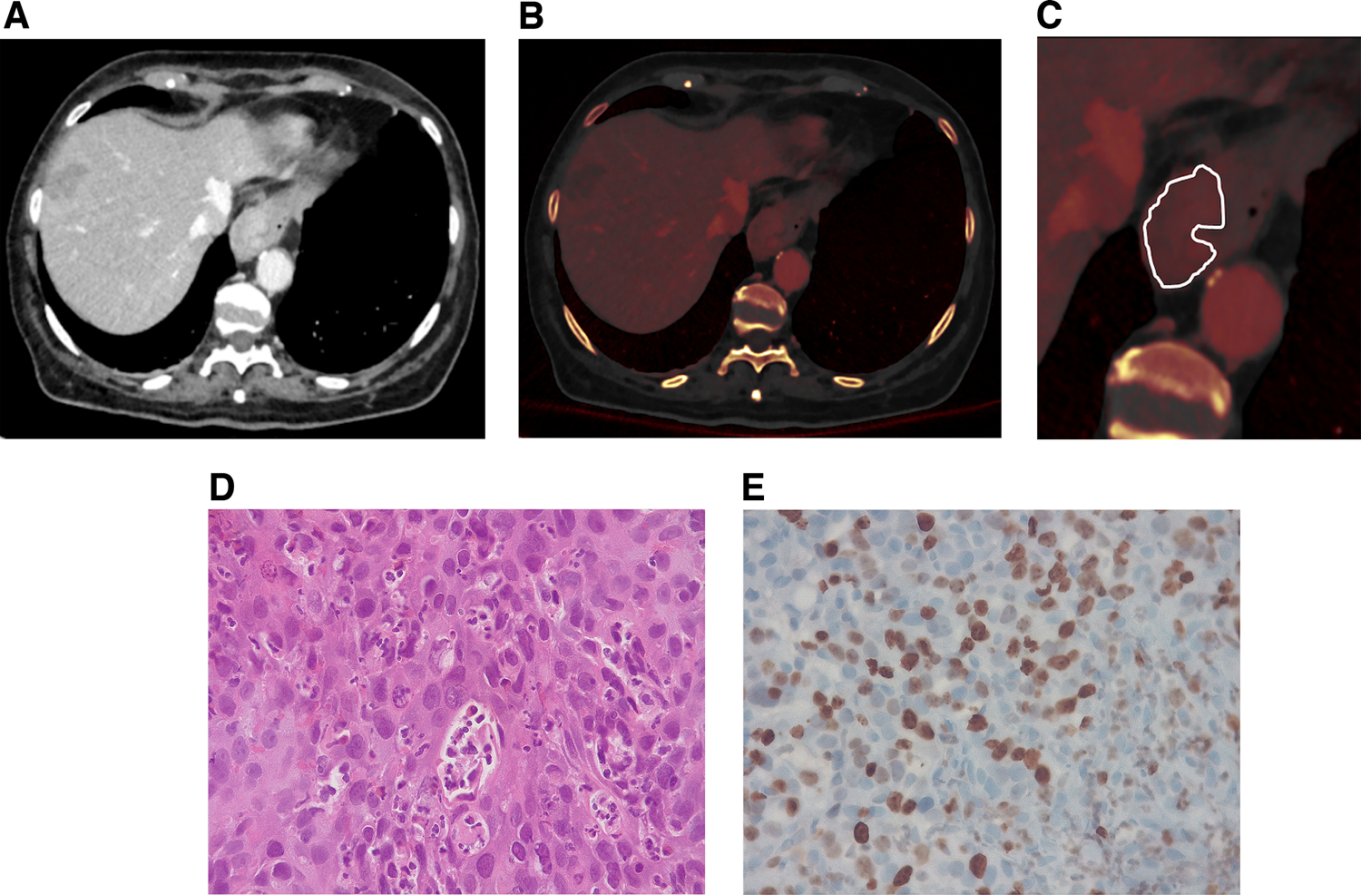
74-year-old female patient with adenocarcinoma of the esophagus T3NxMx, Grade 2. **A** The PCCT scan in the portal venous phase with the corresponding overlaying iodine map (**B**). **C** The tumor is enclosed in a polygonal ROI (white line, magnified view), relative iodine density is 0.47. **D** Hematoxylin-eosin-stained slides of the adenocarcinoma of the esophagus specimen, showing architectural distortion, loss of polarity, and cellular polymorphism, along with centrally captured gland. **E** An antigen-stained specimen using MIB-1 monoclonal antibody finding a total cell count of 271 and a high Ki-67-labeling-index of 40%, suggesting a high-grade tumor. PCCT, photon-counting computed tomography; ROI, region of interest

Figure 2 showcases the ROI placement in the EC on PCCT in the portal venous phase with the corresponding pathology specimen.

### Comparison of NIC values across tumor characteristics

In adenocarcinoma cases, NIC values were analyzed across various tumor characteristics. No statistically significant difference was observed in NIC values between lower-grade tumors (G1/G2) and higher-grade tumors (G3), with a *p*-value of 0.26. Additionally, NIC values did not differ significantly between stages N0 (no nodal involvement) and N+ (nodal involvement), with a *p*-value of 0.11. Similarly, no significant differences were found between early-stage tumors (T1-T2) and advanced tumor stages (T3-T4) with a *p*-value of 0.07, nor between M0 and M+ statuses, with a *p*-value of 0.57. For squamous cell carcinoma, NIC values also showed no significant differences when evaluated across tumor grades, nodal involvement, or metastatic status; see Table 2.

**Table 2:** Association of tumor characteristics and NIC values in esophageal cancer

Parameter	Category	NIC value	*p*-value
Adenocarcinoma			
Tumor grade	Grad 1/2	0.40 ± 0.15	
	Grad 3	0.45 ± 0.15	0.26
Nodal status	N0	0.37 ± 0.14	
	N+	0.45 ± 0.15	0.11
Tumor stage	T1/T2	0.36 ± 0.09	
	T3/4	0.45 ± 0.15	0.07
Metastasis	M0	0.44 ± 0.13	
	M1	0.41 ± 0.18	0.57
Squamous cell carcinoma			
Tumor grade	Grad 1/2	0.69 ± 0.49	
	Grad 3	0.43 ± 0.14	0.07
Nodal status	N0	0.85 ± 0.78	
	N+	0.48 ± 0.16	0.41
Tumor stage	T1/T2	0.46*	
	T3/4	0.54 ± 0.35	0.83
Metastasis	M0	0.61 ± 0.37	
	M1	0.34 ± 0.12	0.07

Values are presented as mean and standard deviation

*NIC* normalized iodine concentration, *T* tumor stage, *N* nodal involvement, *M* distant metastasis

* SD not available

A correlation analysis was conducted to evaluate the relationship between NIC values and histopathological parameters, such as Ki-67 expression and cell count, for both adenocarcinoma and squamous cell carcinoma. In adenocarcinoma cases, NIC values displayed weak, non-significant correlations with Ki-67 (*r* = 0.10, *p* = 0.51) and cell count (*r* = −0.21, *p* = 0.14). Similarly, for squamous cell carcinoma, no statistically significant correlations were observed between NIC values and Ki-67 expression (*r* = −0.32, *p* = 0.18) or cell count (*r* = −0.22, *p* = 0.35).

### Predictive performance of NIC for treatment response in adenocarcinoma

The predictive value of NIC for determining treatment response to NARC was assessed specifically in adenocarcinoma cases. When predicting a good response (Becker grades 1a/1b), NIC achieved an AUC of 0.734. At this threshold, NIC demonstrated a sensitivity of 72.2% and a specificity of 64.7%. The PPV was 68.4%, and the NPV was 68.8%, resulting in an overall accuracy of 68.5%.

**Fig. 3:**
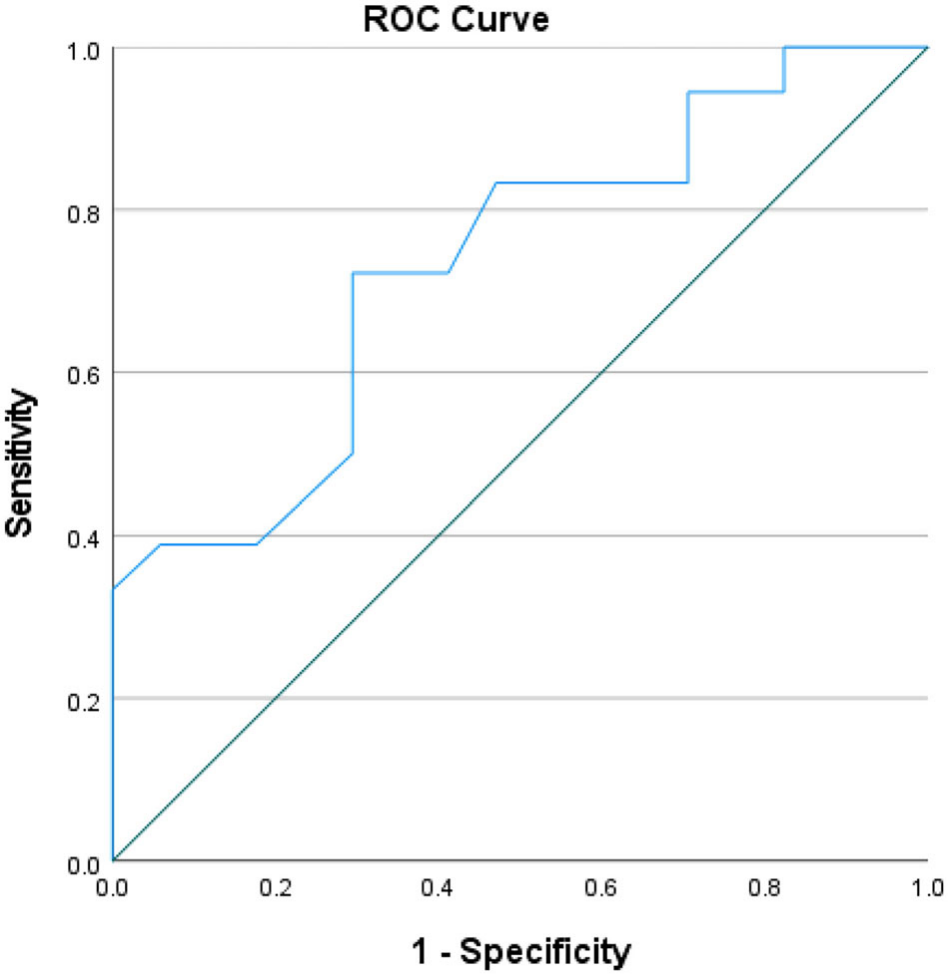
Receiver operating characteristic (ROC) analysis—prognosis of regression in Becker grades 1a and 1b (good prognosis)

**Fig. 4:**
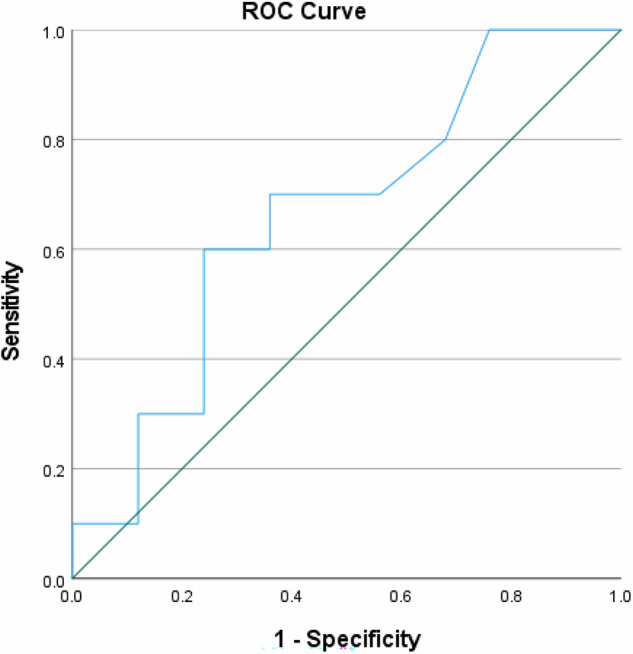
Receiver operating characteristic (ROC) analysis—prognosis of regression in Becker grade 3 (poor prognosis)

For predicting poor treatment response (Becker grade 3), NIC had an AUC of 0.662. Sensitivity for this prediction was 70.0%, with a specificity of 64.0%. The PPV for poor response was 43.8%, while the NPV was 84.2%, leading to an overall accuracy of 65.7%. Figures 3 and 4 present a visual representation of these findings.

An optimal cutoff value for NIC to predict a good treatment response was identified at ≤ 0.41 based on the Youden Index derived from logistic regression, with an AUC of 0.74. At this cutoff, NIC had an OR of 4.77 (95% CI = 1.14–20.00, *p* < 0.01), indicating a relevant association between NIC values at or below this threshold and a favorable treatment response. In the multivariate regression analysis adjusted for tumor stage, nodal involvement, and tumor grade, NIC remained the sole significant parameter associated with good treatment response (AUC 0.79, OR = 3.63, 95% CI = 1.27–10.48, *p* = 0.01).

## Discussion

This study investigated the role of IC derived from PCCT as a prognostic biomarker in EC. The findings revealed that IC demonstrated moderate predictive performance for tumor response to NARC in adenocarcinoma. While NIC did not correlate with histopathological features such as tumor grade or Ki-67 expression, its ability to predict treatment response highlights its potential clinical utility in EC management.

The utility of IC quantification in cancer imaging has been well-documented in other cancers, such as rectal cancer, where it has shown strong associations with treatment response [8]. Our findings align with the growing body of literature supporting iodine-based imaging biomarkers in oncology. For example, Zopfs et al [6] demonstrated that iodine overlays and low-keV virtual monoenergetic images from spectral detector CT improved the qualitative assessment of EC, particularly in delineating tumors and lymph nodes [6]. Although our study focused on treatment response rather than locoregional assessment, both studies underscore the value of iodine-based imaging in enhancing the assessment of EC.

Similarly, Wang et al [7] explored the role of spectral CT in assessing the pathological grade of esophageal SCC, showing that iodine-based imaging could reflect tumor differentiation [7]. Findings from Wang et al [11] and Wu et al [12] further suggest NIC’s potential as a biomarker, correlating it with proliferation marker Ki-67 and molecular subtypes (microsatellite instability vs. microsatellite stability) [11, 12]. However, our findings showed no correlation between NIC and tumor grade or Ki-67 expression. This discrepancy may stem from our focus on treatment response rather than differentiation, as well as the potential underrepresentation of hypovascularized tumors, limiting NIC’s sensitivity.

In their meta-analysis Wang et al [13] evaluated 11 immunohistochemical markers in esophageal SCC with prognostic significance, including vascular endothelial growth factor, Cyclin D1, and p-mTOR, associated with poor outcomes, and P16 and E-cadherin, linked to favorable prognosis. vascular endothelial growth factor, reflecting angiogenesis, and Ki-67, a proliferation marker, are particularly relevant given NIC’s association with vascularity in other cancers [13]. Although this study did not explore molecular insights in detail, combining advanced imaging techniques with molecular profiling could provide a more dynamic and personalized diagnostic approach for EC patients.

Predictive modeling in EC utilizes the combination of imaging and molecular biomarkers to predict treatment response throughout different modalities. These models used, for example, dynamic radiological features from CT could predict pathological response following neoadjuvant immunochemotherapy in SCC, combined MR radiomics and dynamic hematological factors to predict pathological response to NARC, or integrated dynamic contrast-enhanced MRI and diffusion-weighted imaging to predict treatment outcomes [14–16]. Beukinga et al [17] further highlighted the potential of imaging biomarkers for response prediction by using textural features derived from 18F-FDG PET/CT to predict NARC outcomes in EC [17, 18]. These studies underscore the value of non-invasive imaging metrics in treatment response prediction, although they use different imaging modalities. Our study contributes to this field by suggesting that NIC from PCCT could be incorporated into predictive models, potentially improving the accuracy of treatment response predictions for NARC in EC.

This study has several limitations that should be acknowledged. First, its retrospective design may introduce bias, and the lack of qualitative assessment of the PCCT images means that potential outliers in image quality were not detected. Furthermore, no direct comparisons were made between ROI measurements from PCCT and surgical specimen measurements, which could limit the precision of our findings. Finally, the relatively small sample size may have led to the underrepresentation of hypo-enhancing esophageal tumors, which could limit the sensitivity of our proposed method in such cases.

## Conclusion

This study demonstrated that NIC values obtained via PCCT offer promise as an imaging biomarker for predicting treatment response to NARC in esophageal adenocarcinoma. Integrating NIC into dynamic or multi-parametric models holds the potential to increase the granularity of tumor assessment, moving beyond simple anatomical imaging toward a more comprehensive view of tumor biology. As predictive models continue to evolve, the inclusion of NIC could refine treatment planning, allowing for earlier and more accurate identification of patients who are likely to respond favorably to therapy.

## Abbreviations

AUC: Area under the curve
CI: Confidence intervals
EC: Esophageal cancer
IC: Iodine concentration
ICC: Intraclass correlation coefficient
NARC: Neoadjuvant radiochemotherapy
NIC: Normalized iodine concentration
NPV: Negative predictive value
OR: Odds ratios
PCCT: Photon-counting computed tomography
PPV: Positive predictive value
ROC: Receiver operating characteristic curve analysis
ROI: Region of interest
SCC: Squamous cell carcinoma
